# Bioinformatics Analysis Revealed Novel 3′UTR Variants Associated with Intellectual Disability

**DOI:** 10.3390/genes11090998

**Published:** 2020-08-26

**Authors:** Junmeng Yang, Anna Liu, Isabella He, Yongsheng Bai

**Affiliations:** 1 Shanghai Starriver Bilingual School, Shanghai 201100, China; yjm_jasmine@163.com; 2 Appleby College, Oakville, ON L6L3V7, Canada; 2021021@appleby.on.ca; 3 Pittsford Mendon High School, 472 Mendon Road, Pittsford, NY 14534, USA; iheviolin@gmail.com; 4 Department of Biology, Eastern Michigan University, 441 Mark Jefferson Hall, Ypsilanti, MI 48197, USA; 5 Next-Gen Intelligent Science Training, Ann Arbor, MI 48105, USA

**Keywords:** intellectual disability, single nucleotide variants, 3′UTR, miRNA targeting

## Abstract

MicroRNAs (or miRNAs) are short nucleotide sequences (~17–22 bp long) that play important roles in gene regulation through targeting genes in the 3′untranslated regions (UTRs). Variants located in genomic regions might have different biological consequences in changing gene expression. Exonic variants (e.g., coding variant and 3′UTR variant) are often causative of diseases due to their influence on gene product. Variants harbored in the 3′UTR region where miRNAs perform their targeting function could potentially alter the binding relationships for target pairs, which could relate to disease causation. We gathered miRNA–mRNA targeting pairs from published studies and then employed the database of microRNA Target Site single nucleotide variants (SNVs) (dbMTS) to discover novel SNVs within the selected pairs. We identified a total of 183 SNVs for the 114 pairs of accurate miRNA–mRNA targeting pairs selected. Detailed bioinformatics analysis of the three genes with identified variants that were exclusively located in the 3′UTR section indicated their association with intellectual disability (ID). Our result showed an exceptionally high expression of *GPR88* in brain tissues based on GTEx gene expression data, while *WNT7A* expression data were relatively high in brain tissues when compared to other tissues. Motif analysis for the 3′UTR region of *WNT7A* showed that five identified variants were well-conserved across three species (human, mouse, and rat); the motif that contains the variant identified in *GPR88* is significant at the level of the 3′UTR of the human genome. Studies of pathways, protein–protein interactions, and relations to diseases further suggest potential association with intellectual disability of our discovered SNVs. Our results demonstrated that 3′UTR variants could change target interactions of miRNA–mRNA pairs in the context of their association with ID. We plan to automate the methods through developing a bioinformatics pipeline for identifying novel 3′UTR SNVs harbored by miRNA-targeted genes in the future.

## 1. Introduction

Intellectual disability (ID), also called mental retardation (MR) or intellectual developmental disorder (IDD), is defined as a group of developmental conditions characterized by significant impairment of cognitive functions which are associated with limitations of learning, adaptive behavior, and skills [1]. It requires the following criteria for a characterization of ID: (1) deficits in intellectual functioning—“reasoning, problem solving, planning, abstract thinking, judgment, academic learning, and learning from experience”—confirmed by clinical evaluation and individualized standard IQ testing; (2) deficits in adaptive functioning that significantly hamper conforming to developmental and sociocultural standards for the individual’s independence and ability to meet their social responsibility; and (3) the onset of these deficits during childhood [2].

Existing literatures have identified many ID-related genes. For instance, orphan receptors of the rhodopsin (class A) family of G protein-coupled receptors (GPCR) are shown to have significant physiological influences on the mammalian brain and are especially involved in neurodegenerative diseases and psychiatric disorder including Alzheimer’s disease, Parkinson’s disease, neuroinflammation, etc. [3]. In fact, many common kinds of ID are caused by variants in genes. Rett Syndrome, for instance, is demonstrated to be caused by the mutation of *MECP2* gene, where activation is important in the neural maturation process, dendrogenesis and synaptogenesis stimulation, and in the electrophysiological response of the neuron [4]. Similarly, previous studies have found that Fragile X Syndrome, another kind of ID, is caused by the absence of the Fragile X Mental Retardation Protein (FMRP), due to mutations of the *FMR1* gene. In fact, both diseases are oftentimes caused by mutagens in the untranslated regions (UTRs) of the genes: MeCP2 protein expression is regulated by miR-130a targeting, which leads to the inhibition of neurite outgrowth and decrease in dendritic spine density and complexity. The mutation leading to over 98% of cases of fragile X syndrome is an expansion of an unstable CGG repeat sequence located in the 5′UTR of the *FMR1* gene.

During transcription, the expression of coding messenger RNAs (mRNAs) can be targeted and regulated by miRNAs. MiRNAs play a key role in gene regulation, where abnormal function on their target can potentially cause diseases. In mammals, miRNAs are predicted to control the activities of ~60% of all protein-coding genes [5]. As key post-transcriptional regulators, miRNAs have an important role in a wide range of biological processes, including cell proliferation, differentiation, apoptosis, and metabolism as well as pathogenesis of complex diseases, such as cancer and mental disorders [6,7,8].

Genetic variants, such as single nucleotide variations (SNVs), are often thought to contribute to disease development in the context of changing the gene product. Today, sequencing methods such as Next Generation Sequencing (NGS) allow us to study genetic disorders at the level of SNVs. In fact, genetic causes account for 45% of ID [9]. Both whole genome sequencing (WGS) of patients with severe ID and meta-analysis on thousands of de novo mutations demonstrate that candidate ID-causing genes may harbor an excess number of loss-of-function (LoF) or functional de novo mutations [10,11]. However, due to the genetic heterogeneity of ID and its relationship with environmental causes, newly identified genes account for only a small proportion of ID cases [12,13]. Therefore, it is crucial to use available sequencing data to effectively prioritize the causative mutations and candidate genes associated with ID [14]. In terms of the genetic cause, though a majority of cases still remain undiagnosed, microarray studies and exome sequencing have demonstrated the importance of SNVs in ID [13,15,16,17]. The relationship between SNVs and ID is further shown by the entire genome sequencing of 50 patients with severe ID and their unaffected parents. The results suggest that, on average, there are 82 high-confidence potential de novo SNVs identified per genome [10]. Recent studies have found by exome analyses that p.C291X, a homozygous deleterious mutation, is in the *GPR88* gene, contributing to Chorea, a neurotic disease [18]. This suggests one potential connection between *GPR88* and intellectual disability.

In fact, variants that affect noncoding regulatory regions of the genome are related to neurodevelopmental disorders, but the noncoding regions themselves are often overlooked [19]. It is thus suggested that de novo variations in 3′UTR regulatory regions may contribute to ID, specifically when multiple mutations would contribute to a phenotype. For instance, a previous study reported a mutation in *BRCA1* where a functional single nucleotide variant in the 3′UTR can cause decreased *BRCA1* expression, leading to increased breast cancer risk and stage IV breast cancer [20]. Another study identified a novel SNP (rs231725) located in the 3′ flanking region of CTLA4, showing a relatively strong association with primary biliary cirrhosis (PBC) and rs231725, a SNP located outside of the area previously investigated in PBC [21]. Moreover, genome-wide investigation of an ID cohort reveals de novo 3′UTR regulatory variation that affects gene expression and its functionality [22]. Examples such as those suggest the key involvement of miRNAs targeting the commonly-overlooked 3′UTR regions.

Previous studies suggest that numerous single nucleotide polymorphisms (SNPs) within the 3′UTR regions of susceptibility genes can affect complex traits/diseases by influencing the binding affinity of miRNAs [23]. For example, with its putative involvement in the etiology of both neuropsychiatric disorders and cancer, *miR-137* plays an important role in neural development and neoplastic transformation [24]. Mutations related to *miR-137* are causative of 44 diseases according to the Human microRNA Disease Database (HMDD). These diseases include Alzheimer’s disease, carcinoma in multiple tissues, Huntington Disease, Schizophrenia, etc., as well as ID. In fact, *miR-137* is the only miRNA shown to be related to ID according to HMDD. Two of miRNA137’s target genes, *GPR88* and *WNT7A*, are suggested to play a just as important role during neural development through regulation of target genes associated with neural stem cell proliferation and differentiation, identified through luciferase assay [25]. Regulation of 3′-UTR elements from *WNT7A* and *GPR88* by *miR-137* is particularly strong and provides support for the role of post-transcriptional regulation in axon and dendritic growth, maturation, and function [25,26].

As miRNAs interact with their respective miRNA recognition elements (MREs) in the 3′UTR region of transcripts, transfected miRNAs bound to their target MREs and repressed the expression of luciferase, the 3′-UTR of *WNT7A* and *GPR88,* are demonstrated to be responsive [25]. *GPR88* protein is a brain-enriched G protein-coupled receptor with a role in modulating the striatal dopaminergic system [27] and regulating medium spiny neuron excitability [28], while *Wnt7A* regulates dendritic spine growth and synaptic strength [29] and mediates synapse density and numbers and hippocampal network structure [30]. During development, WNT signaling is required for cell proliferation and differentiation, cell polarity generation, and embryonic patterning.

When a single nucleotide variant occurs, there are three likely scenarios in which the mutation would cause damage: if there was originally no miRNA binding to the location of the SNV, the mutation of one nucleotide on the gene may trigger a new binding of miRNA; if there was miRNA(s) binding to the location of the SNV, the gene will either have its original miRNA substituted by the mutated SNV that complementarily matches the gene or lose the miRNA binding.

Thus, SNVs in the 3′UTR of the mRNA can lead to alternations in the binding of miRNAs and thus affect the original miRNAs’ regulation. The dbMTS provides all potential SNVs in the miRNA target seed regions in human 3′UTRs and details their functional predictions and annotations [31]. By checking the known or predicted miRNA–mRNA pairs against the dbMTS, we aim to identify putative SNVs that affect miRNA targeting as well as facilitate the prioritization of their functional importance.

In this project, we conducted bioinformatic analysis to identify novel ID-related mutations harbored in genes that are related to ID. Functional annotation for their targeting miRNAs were also performed.

## 2. Materials and Methods

### 2.1. Input Data Curation from Literature

Though there are several widely accepted miRNA targeting databases available, in order to obtain ID-related miRNA–mRNA pairings, we collected known and predicted pairs of miRNA and target genes from published literatures. After extensive research about known miRNA and mRNA targeting relationships, we obtained a list of miRNA–mRNA pairs from nine published literatures [24,32,33,34,35,36,37]. The list of miRNA–mRNA pairs selected for this study is reported in Table S1.

### 2.2. miRNA-Gene Pair Selection

By manually screening for accurate miRNA–mRNA targeting pairs from related literatures, we obtained a total of 114 pairs. We prioritized the miRNAs that had at least one target gene and sorted them according to their names. We checked for aliases by considering all possible names of the target genes, then substituting the aliases with the official gene names suggested in The National Center for Biotechnology Information (NCBI) (www.ncbi.nlm.nih.gov), which provides access to biomedical and genomic information [38].

### 2.3. dbMTS Database Variant Identification

In order to check the 114 pairs of miRNA and target mRNA genes against dbMTS, we used a customized python script pipeline to generate the mRNA and miRNA candidate input pairs that the dbMTS database accepts. To do so, we converted the mRNA gene names into their Ensembl ID using the mart_export file downloaded from Ensembl Biomart (www.biomart.org) [39]. We converted miRNA names into the correct capitalization and converted pre-miRNA numbers into mature miRNA numbers using hairpin.fa and mature.fa, which were downloaded from miRbase (www.mirbase.org) [40].

To check if the variants we obtained were novel, we checked the IDGenetics database (www.ccgenomics.cn/IDGenetics/), UCSC Genome Browser (genome.ucsc.edu), and dbSNP of NCBI (www.ncbi.nlm.nih.gov/SNP) [41,42,43].

### 2.4. Functional Annotation of Candidate ID Gene Associated with Novel 3′UTR SNVs

In order to determine the protein function of the three genes highly expressed in brain tissues, namely *GPR88, *WNT7A*,* and *CDK6*, we checked the protein–protein interaction (PPI) data, GO annotation, the pathways each gene was involved in, as well as the expression among different tissues.

The protein annotation data of the three proteins were obtained from the STRING database (string-db.org) and UniProt. The PPI data were obtained from the STRING database (www.uniprot.org) [44].

### 2.5. Expression Data and Association with Brain Diseases in Genes Exclusively Expressed in 3′UTR Region

The expression distribution data were obtained from the Genotype-Tissue Expression (GTEx) project (www.gtexportal.org), which provides significant variant and gene associations based on permutations of genes among 53 human body tissues, 13 of which are located in the brain [45]. By normalizing the data by taking the logarithm and then multiplying by ten, we were able to generate a heatmap using R to represent the significantly higher expression of three genes (*GPR88*, *WNT7A*, and *CDK6*) in brain tissues.

### 2.6. Pathway and Brain Diseases Association with of Genes Exclusively Expressed in 3′UTR Region

The pathways each gene was involved in were obtained from the Kyoto Encyclopedia of Genes and Genomes Pathways (KEGG Pathways) (www.genome.jp/kegg/pathway.html), while the diseases that genes were causative to were obtained from KEGG Disease (www.genome.jp/kegg/disease) [46].

### 2.7. Conservation Across Other Species

All three genes, namely *GPR88*, *WNT7A*, and *CDK6*, that are exclusively located in the 3′UTR are conserved across multiple species.

Both *WNT7A* and *GPR88* are suggested to conserve in humans, mice, and rats. We chose mice and rats as representative species and obtained the *WNT7A* and *GPR88* 3′UTR sequences from Ensembl. We checked the 3′UTR sequence as well as the location of the SNV using UCSC Genome Browser, and searched for motifs of a corresponding 3′UTR region within the *GPR88* gene from the MEME Suite Motif Database (meme-suite.org/db/motifs) [47].

We then extracted a 100-nucleotide long sequence, with the variant in the middle, from each *GPR88* gene by the location provided by UCSC Genome Browser, and searched again for motifs among human, mice, and rats using MEME.

We further counted the statistics of the discovered motif in the 3′UTR of the human genome, which was downloaded from Ensembl Biomart. The Fisher exact test was used to determine if the ratio of the MEME-identified motif’s and its reverse-complement’s appearances in the 3′UTR of transcripts to the total number of transcripts was significant.

**Figure 1:**
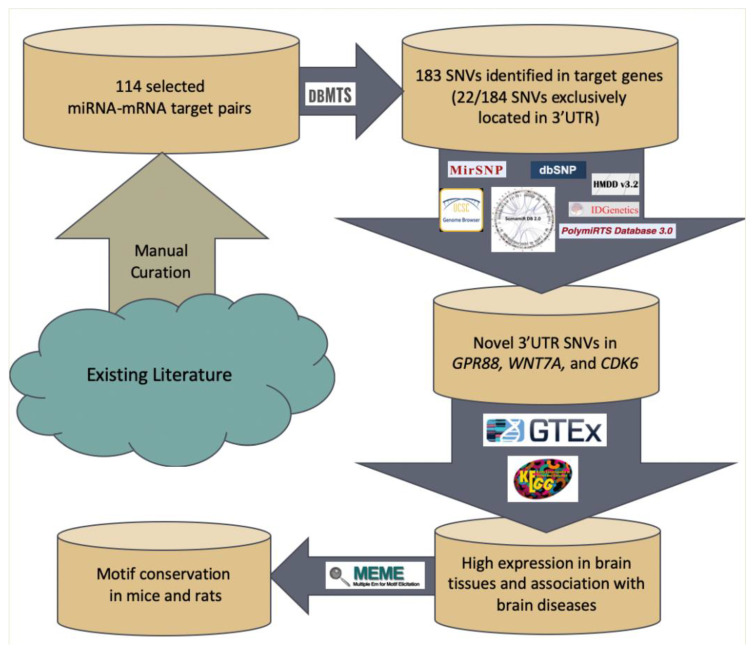
Work flow of prioritizing 3′UTR variants associated with intellectual disability (ID).

Above mentioned steps for selecting miRNA–mRNA target pairs and subsequent bioinformatics analysis are shown in Figure 1.

## 3. Results

### 3.1. miRNA-Gene Pair Selection Screen

By manually screening for predicted and/or experimentally validated pairs of miRNAs and each of their target mRNA, we obtained 18 pairs from the dataset provided in a previous study [36]. Similarly, we obtained 23 pairs from another study [35] as well as 45 genes experimentally validated to be targeted by miR-137 [24]. Genes including *SHROOM3*, *FCER1A*, *DLEC1*, and *PSAPL1* are shown to be targeted by miR-145; genes *ROBO2*, *CHAF1B*, *P2RY22*, *XRRA1*, *GPX8*, *P2RY2*, and *NSG1* are targeted by *miR-183* [33]. Genes *ARMCX2*, *NEFH*, and *FMR1* are shown to be targeted by *miR-222* [37]. The gene *MECP2*, which is related to IDs such as Rett Syndrome can be targeted by *miR-302c*, *miR-483–5p*, *miR-130a*, and *miR-200a*. The pair of *miR-128* and *PHF6* [34] and the pair of *miR-155* and *AGTR1* [32] are similarly obtained.

The list of miRNA–mRNA pairs selected for this study is reported in Table S2.

### 3.2. Analysis of Result from dbMTS

Upon checking our 114 miRNA and target gene pairs against the dbMTS database, we obtained 183 SNVs that were present in the genes and could potentially affect miRNA binding. In the 183 SNVs we obtained through checking the original miRNA-gene pairs against dbMTS, 180 of them experienced a loss in miRNA targeting, and 161 SNVs out of 183 SNVs were matched with alternative miRNAs. Although dbMTS reports all SNVs for single reference positions, some cases could fall into different genomic classification regions. Variants could be specific for different isoforms. A total of 22 of the SNVs were exclusively located in the 3′UTR of all possible transcripts. Those 22 highly confident 3′UTR SNVs were harbored within three genes: *GPR88*, *WNT7A*, and *CDK6*, as shown in Table 1. The full results generated by dbMTS can be found in Table S2.

### 3.3. Novel SNV Identification

With dbMTS, we discovered novel variants that were previously not reported by literatures as well as databases, including the UCSC Genome Browser, IDGenetics database, and dbSNP of NCBI. For instance, the SNV in *GPR88*, which is potentially an ID-causing SNV, has been neither reported in databases nor by literatures. In order to confirm the novelty of the SNVs, we also checked the database mirSNP (http://bioinfo.bjmu.edu.cn/mirsnp/search/) and PolymiRTS (http://compbio.uthsc.edu/miRSNP/).

MirSNP, which provides SNPs located in predicted miRNA target sites and is comprised over 414,510 predicted miRNA-related SNPs, does not provide the SNV we obtained from dbMTS for both *CDK6* and *GPR88*, nor does it provide any SNV for the *WNT7A*-mir137 targeting relationship. Neither does PolymiRTS, which provides four times more miRNA-related SNPs than Mirsnpscore and three times more miRNA-related SNPs than Patrocles, provide this SNV.

### 3.4. Functional Annotation of Candidate ID Gene Associated with Novel 3′UTR SNVs

**Figure 2:**
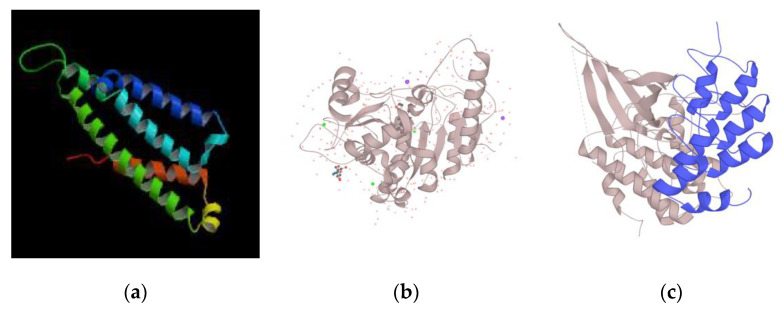
Protein Structure of genes *GPR88*, *WNT7A*, and *CDK6*. (**a**) *GPR88* protein structure predicted by ModBase. (**b**) The *WNT7A* protein structure obtained by X-Ray is shown. The image is provided by from Universal Protein Resource Knowledge Base. (**c**) The 3D structure of *CDK6* obtained by X-ray, is shown, also provided by UniProt. (UniProtKB) [48].

As shown in Figure 2, the protein structure of *CDK6* and *WNT7A* was obtained from the Universal Protein Resource (UniProt), while the predicted protein structure of *GPR88* was obtained from the Database of Comparative Protein Structure Model (ModBase) (https://modbase.compbio.ucsf.edu/), since it is not provided by UniProt.

**Figure 3:**
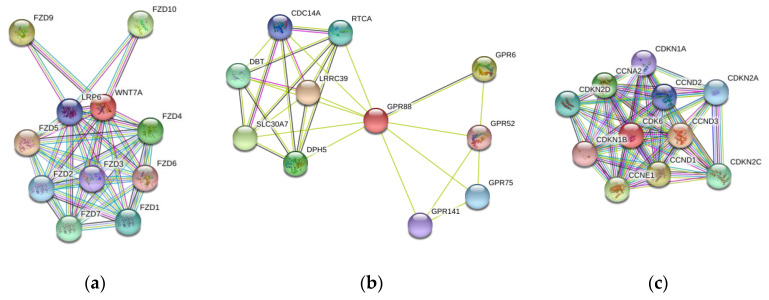
Protein–protein Interaction Data of *GPR88*, *WNT7A*, and *CDK6*. (**a**) Protein *GPR88* interacts with proteins of the same family (i.e., rhodopsin (class A) family of GPCR). (**b**) Protein *WNT7A* interacts with receptor for Wnt proteins as well as component of the Wnt-Fzd-LRP5-LRP6 complex (**c**) Protein *CDK6* interacts with G1/S-specific cyclin, cyclin-dependent kinase inhibitor, and Cyclin.

The PPI data of the three genes shown were obtained from the STRING Database by Figure 3. The protein *GPR88* interacted with proteins of the same family (i.e., rhodopsin (class A) family of GPCR), namely *GPR6*, *GPR52*, *GPR75*, and *GPR141*. Protein *WNT7A* interacted with receptors for Wnt proteins, which were *FZD5*, *FZD9*, *FZD10*, *FZD4*, *FZD7*, *FZD1*, *FZD2*, *FZD3*, and *FZD6*, as well as a component of the Wnt-Fzd-LRP5-LRP6 complex. Protein *CDK6* was suggested to interact with G1/S-specific cyclin, including *CCND3*, *CCND1*, *CCND2*, and *CCNE1*; cyclin-dependent kinase inhibitor, namely *CDKN2C*, *CDKN2D*, *CDKN2A*, and *CDKN1A*; and cyclin *CCNA2*.

### 3.5. Expression Data of Genes Exclusively Expressed in 3′UTR Region

**Figure 4:**
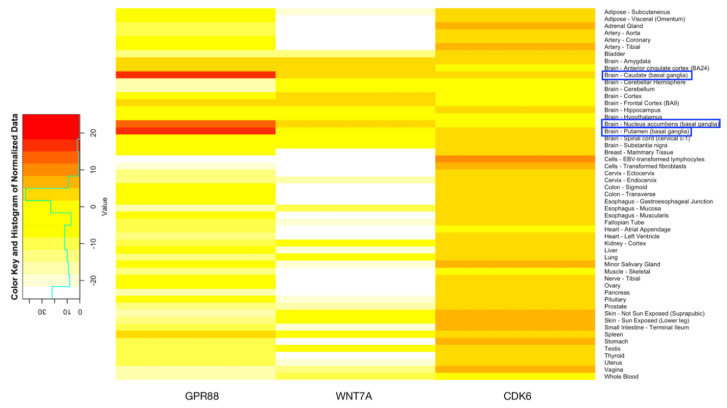
Expression data of the genes *GPR88*, *WNT7A*, and *CDK6* obtained from GTEx.

We obtained expression distribution data, provided in Genotype-Tissue Expression (GTEx), on 53 tissues as shown in Figure 4. *GPR88* was overexpressed in tissues including Caudate, Nucleus accumbens, and Putamen, all of which are in the brain, while the gene *WNT7A* was overexpressed in Amygdala, Anterior cingulate cortex, Caudate, Cortex, Frontal Cortex, and Nucelus accumbens of the brain.

On the other hand, *CDK6*, due its ubiquity, was not significantly expressed in the brain. In fact, *GPR88* had extremely high expressions in brain tissues including caudate (basal ganglia) and putamen (basal ganglia), which suggests a strong connection with brain and neurotic function, potentially related to ID. In addition, the four datasets (HPA RNA-seq normal tissues, RNA sequencing of total RNA from 20 human tissues, tissue-specific circular RNA induction during human fetal development, and Illumina bodyMap2 transcriptome) are suggested in NCBI (www.ncbi.nlm.nih.gov/gene/), which provides expression information among different tissues that the expression of *GPR88* is significantly higher in the brain, further corroborating its relation with ID [38,49,50,51].

A complete list of miRNA targeted genes with GTEx brain tissue expression is reported in Table S3.

### 3.6. Pathway and Brain Diseases Association with Genes Exclusively Expressed in 3′UTR Region

Alongside the Gene Ontology (GO) terms obtained from both UniProt and suggested by STRING database, the pathways and related diseases provided by KEGG further suggested that *GPR88* and *WNT7A* are related to ID. The GO analysis of *GPR88* suggested that the mRNA is involved the molecular function G-protein coupled activity as well as multicellular organism development. The gene was also shown to be the only related gene to the disease Chorea, childhood-onset, with psychomotor retardation, by KEGG. Indeed, according to previous studies [18], *GPR88* deficiency in humans is theorized to manifest as developmental delay with pronounced speech acquisition impairment, learning disabilities, and hyperkinetic movement disorder at 8–9 years of age.

*WNT7A* was shown to be involved in all three GO functional enrichments, namely biological processes regarding the Wnt signaling pathway, molecular function in terms of Wnt-protein, and Wnt-activated receptor binding and Wnt-protein binding, and cellular component in/on the cell and vesical membranes. KEGG suggested that the Wnt signaling pathway is related to Alzheimer’s disease, while the mTOR signaling pathway, which the protein is also involved in, is causative to diseases such as autism and epilepsy, which are related to ID.

On the other hand, *CDK6* was suggested to interact with Cyclin-dependent kinase 4 inhibitor and Regulatory component of the cyclin. All biological process, molecular function, and cellular component of GO functional enrichment suggested by UniProt are related to the cell cycle, further indicating the fact that it is a ubiquitous gene and is more related to cancer.

### 3.7. Conservation across Other Species

All biological process, molecular function, and cellular component of GO functional enrichment of all three genes, namely *GPR88*, *WNT7A*, and *CDK6*, are conserved in multiple species. *GPR88*, for instance, is conserved in 28 species in total according to UniProt, while KEGG Organisms of NCBI Taxonomy suggests a total of 109 species. There were 16 SNVs reported by dbMTS that were located in *WNT7A*. Fifteen of them were of the positions 13818646 to 13818650, and those five nucleotides were found to be conserved among humans, mice, and rats by MEME.

**Figure 5:**
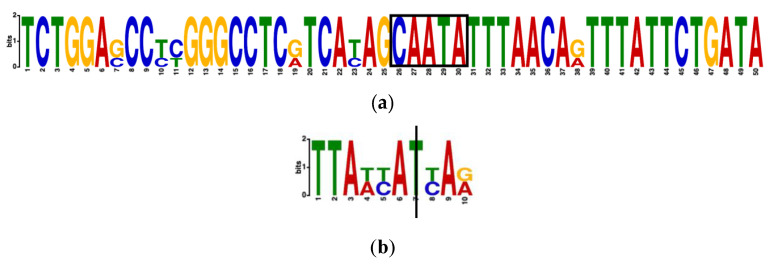
Motif conservation of genes *GPR88* and *WNT7A* among humans, mice, and rats. Each graph obtained from MEME represents a motif discovered. (**a**) The motif in *WNT7A* 50 nucleotides long, with the boxed nucleotides at position 13818650–13818646 in reverse complement order. (**b**) The 10-nucleotide long motif containing the SNV at position 100541492 is in *GPR88*, conserved only among humans and mice.

MEME identified many motifs of the gene *WNT7A* in humans, mice, and rats. Among them, the motif that contained five out of the six SNVs in *WNT7A* that were identified by dbMTS was the third most significant. With an *E*-value, which MEME adopted to present the statistical significance of the motif of 2.1 × 10^−10^, the motif ranged from 13818626 to 13818675 in the human genome, with the five neucleotides TATTG, which were in reverse-complement order since the template strand of *WNT7A* in humans was on the negative strand, in the middle. In addition, the human *WNT7A* gene had a *p*-value of 4.69 × 10^−29^ to the motif. Figure 5a shows the five neucleotides as boxed.

The MEME search result did not give a significant motif that included the variant identified by dbMTS in *GPR88* genes in humans, mice, and rats. The variant, however, was found to be conserved among humans and mice. By conducting a motif search using three sequences of 100-nucleotides long with the identified variant in the middle, the motif containing the variant, which was conserved between human and mouse, had a *E*-value of 8.6 × 10^4^. The motif included the variant identified by dbMTS, which was at location 100541492 in humans (the third nucleotide to the left, T), was conserved. The *GPR88* motif (TTAACATCAA) presence test for our three identified ID related genes at the level of 3′UTR region in human was significant (Fisher exact test statistic value is 0.0218).

The human *WNT7A* sequence had a *p*-value of 1.61 × 10^−3^. The motif 20 nucleotides to the left of the identified SNV had a *p*-value of 2.85 × 10^−9^; the motif five nucleotides to the right of the identified SNV had a *p*-value of 91.43 × 10^−21^. The motif conservation in mice is shown in Figure 5b, with the vertical bar representing the location of the SNV.

## 4. Discussion

Of the 183 SNVs we obtained through checking the original miRNA-gene pairs against dbMTS, 180 of them are in the genes of the input pairs that are successfully matched with reference miRNA-gene pairs in dbMTS. This indicates that these 180 SNVs are validated in terms of having either target losses or substitutions of miRNA. On the other hand, 162 SNVs out of 183 SNVs are in genes of the input pairs that are matched with the alternative pairs, suggesting a gain in miRNA targeting. Additionally, in terms of the three mRNAs that have SNVs exclusively expressed in the 3′UTR, all 22 SNVs that are found in those three genes, except for *GPR88* at position 100541492, have a miRNA target loss, while all 22 SNVs except for *WNT7A* at position 13818646 and 13818647, as well as *CDK6* at position 92237307, experience a target gain.

All three genes in which dbMTS identified SNVs were reported to be targeted by miR-137.

Through luciferase assay, researchers have identified the targets from the 3′UTR of *WNT7A* and *GPR88* as most responsive in a previous experiment consisting of multiple genes sequences, including other targets of miR-137, supporting the existence of relationships between genes reported to be associated with neural development and developmentally regulated miRNAs. As an orphan GPCR protein, *GPR88* plays an important role in psychopharmacology and is a potential new target for the treatment of major CNS diseases. *GPR88* has a crucial role in dopamine neurotransmission and striatal physiology [52]. Previous studies suggest that *GPR88* is involved in schizophrenia [53], is related to a major psychosis in triads from the Xhosa population, has a positive association with bipolar disorder in the Sardinian and Palestinian triads, [54] and is causative to chorea, which further affects speech delay and learning disabilities [18]. *WNT7A* is involved in dendritic growth, since its signaling is critical to dendritic spine growth and synaptic strength regulation [29], synapse density and amount mediation [30], etc. Meanwhile, it also has a significant impact on macrophage responses by inducing a unique phenotype of monocyte-derived macrophages [55], and is related to embryo development and tissue homeostasis as it can initiate both β-catenin dependent and independent pathways [56]. The basal ganglia, part of the subcortical structures of the brain, is involved in memory, emotion, pleasure, and hormone production. A research study [57] about the most frequent mutation found in the *ARX* gene suggests that the *ARX* gene is responsible for XLAG syndrome and milder forms of X-linked ID. Not only is the basal ganglia known to regulate sensorimotor processing, ARX patients also have a significantly decreased volume of brain structures including the nucleus of caudate, in which *GPR88* and *WNT7A* overexpresses; as shown in Figure 4.

CDK6 is a validated target in the treatment of Glioblastoma, a kind of brain tumor that is characterized by a high frequency of CDKN2A/CCND2/CDK4/CDK6 pathway dysregulation [58]. In fact, targeting cancer-related genes such as *CDK6* and *CDC43*, miR-137s is involved not only in the regulation of transition from pluripotentency to differentiated states but also in apoptosis, suggesting that it is also associated with cancer. A role for miR-137 as a tumor suppressor is evident: overexpression of miR-137 was reported to decrease the expression of *CDK6*, suggesting that miR-137 acts through the inhibition of *CDK6* in lung cancer cells, while the decreases in miR-137 expression in tumor cells support the role of miR-137 in the pathogenesis of cancer, including pancreatic cancer, 109 osteosarcoma cancers, 110 gastric cancers, 48 oral cancers, and 50 ovarian cancers [24]. Protein expression levels of *CDK6*, as well as *E2F6* and *NCOA2/TIF2*, were clearly reduced in all miR-137 transfectants compared with their control counterparts [26]. In addition, *CDK6* is also a predicted target miR-124 [59].

The GTEx expression as well as KEGG Pathway both indicate that *GPR88* is highly related to intellectual disability, as *GPR88* has extremely high expression among brain tissues compared to the other tissues and other genes that are overexpressed in brain tissues. For *WNT7A*, though expression in brain tissues is not as high as that of *GPR88*, it is still significant compared to other tissues. Predictably, due to its ubiquitous expression across a large number of tissues, *CDK6* is not shown to be overexpressed in the brain, possibly suggesting that its relationship to Intellectual Disability is not as significant compared to other diseases, most of which are cancer.

## 5. Conclusions

Existing literatures have reported miRNAs and their target genes associated with ID, but genetic variants located in the 3′UTR region have not been elucidated for the target relationship creation and/or disruption. Using dbMTS, we discovered novel variants in the 3′UTR of genes associated with intellectual disability and targeted by miRNAs. *GPR88*, which contains a variant and is exclusively expressed in the 3′UTR, is found to have an exceptionally high expression in brain tissues. Containing 16 SNVs obtained from dbMTS, *WNT7A* conserves in multiple species and has significant motifs discovered by MEME among the human, mouse, and rat sequences. The third most significant motif discovered includes five out of six positions of SNVs discovered by dbMTS. GO annotations and pathways further suggest that the two genes are related to multiple nervous system diseases. For instance, *GPR88* was reported to be causative of Chorea with psychomotor retardation. Our study provides the process of prioritizing SNVs that are likely to contribute to miRNA–mRNA target relationship associated with diseases. It would be valuable to develop a bioinformatics pipeline for identifying novel 3′UTR SNVs harbored by miRNA-targeted genes. Future experimental validation can be performed to validate if our identified novel 3′UTR variants for *GPR88* and *WNT7A* serve as the miRNAs binding sites.

## Figures and Tables

**Table 1:** Identified variants located in 3′UTR regions that alter miRNA targeting based on dbMTS.

Chr	Pos	Ref	Alt	VEP_Ensembl_Gene_Name
1	100541492	T	G	*GPR88*
3	13818428	G	T	*WNT7A*
3	13818646	T	A	*WNT7A*
3	13818646	T	C	*WNT7A*
3	13818646	T	G	*WNT7A*
3	13818647	A	C	*WNT7A*
3	13818647	A	G	*WNT7A*
3	13818647	A	T	*WNT7A*
3	13818648	T	A	*WNT7A*
3	13818648	T	C	*WNT7A*
3	13818648	T	G	*WNT7A*
3	13818649	T	A	*WNT7A*
3	13818649	T	C	*WNT7A*
3	13818649	T	G	*WNT7A*
3	13818650	G	A	*WNT7A*
3	13818650	G	C	*WNT7A*
3	13818650	G	T	*WNT7A*
7	92605125	T	A	*CDK6*
7	92607925	A	G	*CDK6*
7	92607993	A	C	*CDK6*
7	92607993	A	G	*CDK6*
7	92607993	A	T	*CDK6*

Note: The first column, “chr”, indicates the chromosome number where the SNV is found; the second column, “pos”, refers to the position of each SNV on the chromosome. The third and fourth column, “ref” and “alt”, show reference nucleotide and the mutant nucleotide, respectively, and thereby present the single nucleotide variant. The last column, “VEP_ensembl_Gene_Name”, indicates the gene name in which the SNV occurs.

## Supplementary Materials

The following are available online at https://www.mdpi.com/2073-4425/11/9/998/s1. Table S1: Curated MiRNA–mRNA target pairs list. Table S2: dbMTS result of miRNA–mRNA target pairs. Table S3: GTEx Gene expression data of genes *GPR88*, *WNT7A*, and *CDK6* across 53 tissues.

## Abbreviations

ID	Intellectual disability
miR	MicroRNA
UTR	Untranslated region
SNV	Single variant nucleotide
GPCR	G protein-coupled receptors
PPI	Protein-protein interactions
